# The Study of Interfacial Adsorption Behavior for Hydroxyl-Substituted Alkylbenzene Sulfonates by Interfacial Tension Relaxation Method

**DOI:** 10.3390/molecules28114318

**Published:** 2023-05-24

**Authors:** Qi Sun, Zhicheng Xu, Qingtao Gong, Wangjing Ma, Zhiqiang Jin, Lei Zhang, Lu Zhang

**Affiliations:** 1 Key Laboratory of Photochemical Conversion and Optoelectronic Materials, Technical Institute of Physics and Chemistry, Chinese Academy of Sciences, Beijing 100190, China; qisun2020@163.com (Q.S.); zhchxu@mail.ipc.ac.cn (Z.X.); gongqt@mail.ipc.ac.cn (Q.G.); wjma@mail.ipc.ac.cn (W.M.); jinzhiqiang@mail.ipc.ac.cn (Z.J.); 2 University of Chinese Academy of Sciences, Beijing 100049, China

**Keywords:** hydroxyl-substituted alkylbenzene sulfonate, interfacial tension relaxation method, dilational elasticity, dilational viscosity, surface adsorption, adsorption

## Abstract

In order to explore the interface adsorption mechanism of hydroxyl-substituted alkylbenzene sulfonates, the interfacial tension relaxation method was used to investigate the dilational rheology properties of sodium 2-hydroxy-3-octyl-5-octylbenzene sulfonate (C_8_C_8_OHphSO_3_Na) and sodium 2-hydroxy-3-octyl-5-decylbenzene sulfonate (C_8_C_10_OHphSO_3_Na) at the gas–liquid interface and oil–water interface. The effect of the length of the hydroxyl para-alkyl chain on the interfacial behavior of the surfactant molecules was investigated, and the main controlling factors of the interfacial film properties under different conditions were obtained. The experimental results show that for the gas–liquid interface, the long-chain alkyl groups adjacent to the hydroxyl group in the hydroxyl-substituted alkylbenzene sulfonate molecules tend to extend along the interface, showing strong intermolecular interaction, which is the main reason why the dilational viscoelasticity of the surface film is higher than that of ordinary alkylbenzene sulfonates. The length of the para-alkyl chain has little effect on the viscoelastic modulus. With the increase in surfactant concentration, the adjacent alkyl chain also began to extend into the air, and the factors controlling the properties of the interfacial film changed from interfacial rearrangement to diffusion exchange. For the oil–water interface, the presence of oil molecules will hinder the interface tiling of the hydroxyl-protic alkyl, and the dilational viscoelasticity of C_8_C_8_ and C_8_C_10_ will be greatly reduced relative to the surface. The main factor controlling the properties of the interfacial film is the diffusion exchange of surfactant molecules between the bulk phase and the interface from the beginning.

## 1. Introduction

Surfactant is a kind of unique compound. The addition of a small amount of surfactant in the system can change the interface composition and structure and greatly reduce the surface/interfacial tension [1]. In recent years, with the continuous progress of the discipline of colloid and interface chemistry, people have no longer only paid attention to the ability of surfactants to reduce interfacial tension but have also begun to fully explore their properties in emulsification, wetting, foaming, solubilization, catalysis, anticorrosion and other aspects [2,3,4,5], resulting in energy saving and consumption reduction [6], biochemical detection [7], food production [8], oil exploitation [9,10] and benefits in other fields, which has brought great convenience to human production and life. As a typical anionic surfactant, alkylbenzene sulfonate is favored for use in oil fields due to its low price and wide source. At the same time, the introduction of a suitable substitution structure on benzene ring can also promote the stability of foam or emulsion [11,12]. Therefore, this kind of surfactant has become one of the most commonly used oil displacement agents in tertiary oil recovery technology over the past ten years [13,14].

When different kinds of surfactant molecules spontaneously enrich at the interface, there are differences in the number and state of adsorption. The former determines the value of the interfacial tension and the latter is closely related to the strength of the interfacial film. The interfacial tension can be directly measured by the Wilhelmy plate method [15,16,17], the rotating drop method [9,18,19,20] and other methods. However, the characterization of interfacial film strength is relatively complex. In order to avoid the interference of liquid film thickness, bulk viscosity and other factors, it is necessary to use dilational rheology [21,22,23]. The interfacial dilational rheology test chooses to apply a periodic sinusoidal disturbance to the droplet or liquid surface to make it expand and compress continuously. The strength of the interfacial film is measured by the change of the interfacial tension with the interfacial area during the dynamic process; that is, the dilational modulus. The larger the modulus, the higher the strength. The dilational modulus is composed of elastic modulus and viscous modulus. The elastic modulus represents the magnitude of the interaction between surfactant molecules at the interface, while the viscous modulus is the sum of all relaxation processes occurring at and near the interface, including the diffusion and exchange between surfactant molecules and bulk phase, the interfacial rearrangement of surfactant molecules, etc. [11,24,25,26].

The appearance of interfacial dilational rheology makes it possible to accurately divide the strength and free energy of interfacial films. Unfortunately, due to the upper limit of the oscillator, the existing dilational rheology experiments are dominated by small low-frequencies, and the oscillation frequency generally does not exceed 1 Hz [27,28,29]. It should be pointed out that for the fast relaxation processes such as diffusion exchange and reorientation of single molecules on the interface, the working frequency of 1 Hz is far from enough. At this time, after the external work on the interface film, the recovery speed of the film composition is ahead of the change speed of the interface area, which is not conducive to the full acquisition of information in the film. In this case, the interfacial tension relaxation method, as an important part of the interfacial dilational rheology, perfectly compensates for the shortcomings of the periodic oscillation method. By instantaneously expanding a fully pre-balanced interface in a short time and then recording and multi-exponentially fitting the decay process of the interfacial tension, the interfacial tension decay curve is transformed by Fourier transform in the time domain to obtain the interfacial dilational viscoelastic parameters in the frequency domain; that is, the full-frequency spectrum of dilational viscoelasticity is obtained [30,31].

The interfacial dilational rheology test of alkylbenzene sulfonates has been carried out for a long time, especially for sodium alkylbenzene sulfonates with different alkyl substitutions and branched structures [32,33,34,35,36]. Song et al. [37] explored the dilational rheological properties of three branched alkylbenzene sulfonate adsorption films at the gas–liquid interface by using the periodic oscillation method, and they found that the position of the benzene ring in the alkyl chain was one of the main factors controlling the properties of the interface film. When the benzene ring gradually moves from the end position to the more central position, that is, the degree of branching of the hydrophobic alkyl chain gradually increases, the dilational elasticity increases and the stability of the interfacial film also increases. Zhu et al. [12] investigated the adsorption behavior of polysubstituted alkylbenzene sulfonates at the n-decane-water interface by interfacial tension relaxation method, and they clarified the main controlling factors of interfacial film properties with a Cole–Cole diagram. They believe that when there is no electrolyte, alkylbenzene sulfonate molecules are tiled on the oil–water interface, and that the interaction is weak. The behavior of the interface film responding to the change of the interface area is a multi-relaxation process such as diffusion exchange, reorientation and interface rearrangement. With the addition of Ca^2+^ and Mg^2+^, under the action of electrostatic shielding, the surfactant molecules began to be more upright at the interface, and the interaction gradually increased. At this time, the intrafilm relaxation process dominated by reorientation controlled the properties of the interfacial film.

In contrast, there are relatively few studies on the interfacial dilational rheology of hydroxyl-substituted alkylbenzene sulfonates on the benzene ring. In our previous work [38,39], we explored the interfacial dynamic dilational modulus of sodium 2-hydroxy-3,5-long-chain alkylbenzene sulfonate by using a small low-frequency oscillation method, and we found that the molecular rearrangement at the interface led to the extreme value of the dynamic modulus. At the same time, the “positioning” effect of the multi-hydrophilic group on the benzene ring causes the entanglement between the substituted alkyl groups, the interfacial film strength is significantly enhanced and the modulus value is much higher than that of ordinary alkylbenzene sulfonates [11]. In this paper, we further studied the adsorption behavior of hydroxyl-substituted alkylbenzene sulfonates at the oil–water interface and the gas–liquid interface by using the interfacial tension relaxation method. The supplement of the full-frequency spectrum of dilatational viscoelasticity enriches people’s understanding of the interfacial behavior of surfactant molecules, which is the theoretical basis of surfactant as a foaming agent and emulsifier, and provides more reliable guidance for the field application of alkylbenzene sulfonate anionic surfactants as chemical flooding formulations.

## 2. Results and Discussion

### 2.1. Full-Frequency Spectrum of Interfacial Dilational Elasticity of Adsorption Film

**Figure 1:**
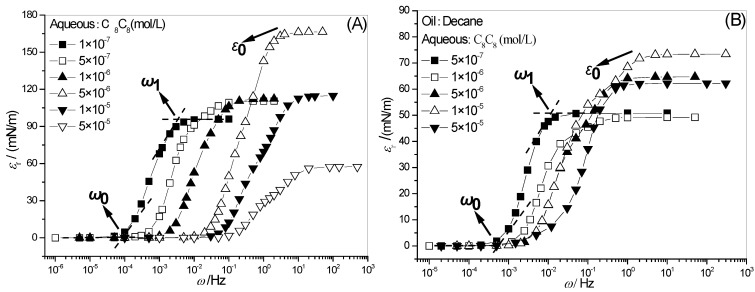
Frequency spectra of surface (**A**) and interfacial dilational elasticity (**B**) for C_8_C_8_ solutions.

Interfacial dilational elasticity is a quantitative description of the interaction strength of surfactant molecules at the interface. Figure 1 shows the full-frequency spectra of the dilational elasticity of hydroxyl-substituted alkylbenzene sulfonate C_8_C_8_ with different concentrations, where (A) is the gas–liquid interface and (B) is the oil–water interface. As the frequency increases, the dynamic modulus curve gradually increases and finally reaches a plateau value, representing the whole process of the state of the interface film from full recovery to untimely change. The characteristics of the curve are described by the frequency *ω*_1_ at which the elasticity begins to increase and the frequency *ω*_0_ that does not change, as shown in Figure 1. At the same time, the concept of limit dilational elasticity *ε*_0_ is introduced here.

For the adsorption film, the interfacial behavior of surfactant molecules on the film in response to external disturbances is mainly divided into two types: escaping into the aqueous phase or changing the average occupied area of surfactant molecules on the interface. For the first type, the impact of the outside world on the interfacial film will be released by diffusion exchange, and there will be no energy storage in the film; the dilational modulus value is small, such as the interfacial film composed of small molecule extended surfactants or cationic surfactants [40,41]. For the second type, whether it is to shorten the distance between each other by winding the molecular chains or to increase the amount of adsorption on the interface by changing the molecular orientation, the main body of the diffusion and exchange between the interface and the bulk phase becomes the solvent molecules. The impact of the outside world is stored in the film in the form of energy and converted into elasticity; the modulus value is higher, such as the interface film composed of betaine surfactants [42,43,44].

(1) $$\varepsilon_{0}=-\Gamma \frac{d\gamma }{d\Gamma }$$

Therefore, under extreme conditions, the deformation imposed by the outside world is slow enough, and the diffusion exchange process occurs fully during the deformation process. The interface composition after deformation is the same as that before deformation, and the elasticity is zero. If the deformation is fast enough, there is no molecular exchange between the interface and the bulk phase, and the external work will be completely converted into the elasticity of the film. The dilational elasticity at this time is called the limit dilational elasticity *ε*_0_, which quantitatively characterizes the degree of change in the interaction force when the number of interfacial molecules (Γ) changes, as shown in Formula (1) [12].

### 2.2. Effect of Surfactant Concentration on ω_1_

**Figure 2:**
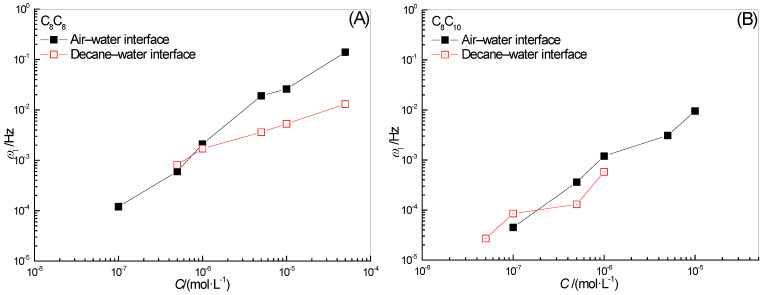
Concentration dependence of $\omega_{1}$ for surfactant solutions at water–air (**A**) and water–decane interfaces (**B**).

The concentration is one of the important factors affecting the properties of the interfacial film. The effects of surfactant C_8_C_8_ and C_8_C_10_ concentrations on *ω*_1_ in the full-frequency spectrum of the surface-interface dilational elasticity are shown in Figure 2. The increase in elasticity means that the composition of the interface film begins to respond to the interface disturbance. At this time, the diffusion exchange between the bulk phase and the interface cannot completely eliminate the influence of the interface deformation. For the absolute value of *ω*_1_, the lower the frequency at which elasticity begins to appear indicates that even at a very low oscillation frequency, the interfacial film still cannot return to its original state as soon as possible, and diffusion exchange is not the main relaxation process. The recovery speed of the interfacial film is not only a direct description of the film-forming ability of surfactant molecules, but is also a side effect of the desorption rate on the film, which is a manifestation of high film strength. On the contrary, the higher the frequency of elasticity begins to appear, the easier it is for the system to quickly return to its original state after the disturbance, the difficulty of diffusion exchange is very small and the surfactant molecules on the interface film can be desorbed at will.

The value of *ω*_1_ of two hydroxyl-substituted alkylbenzene sulfonates increases with the increase in concentration, whether at the gas–liquid interface or the oil–water interface. The higher the concentration, the faster the diffusion exchange process and the higher the corresponding frequency value, which is reasonable. In addition, for Figure 2, *ω*_1_ at the gas–liquid interface is significantly higher than that at the oil–water interface in the entire experimental concentration range. This difference should be caused by the difference in the arrangement of surfactant molecules at different types of interfaces. At the gas–liquid interface, the hydroxyl and sulfonic acid groups on the benzene ring have good water solubility, they exist in the benzene ring in an adjacent location manner, which will have a certain “positioning” effect on the interface orientation of the entire molecule. The long-chain alkyl at the ortho position of the hydroxyl group tends to extend along the interface, while the long-chain alkyl at the para position of the hydroxyl group tends to extend into the air. However, at the oil–water interface, the presence of oil molecules will destroy the van der Waals force between the hydroxyl groups, and the two alkyl chains on the hydrophobic side begin to tend to stretch upright in the oil phase. Surprisingly, for the two surfactants selected in this paper, the interaction between n-decane and hydrophobic alkyl chains has a more obvious inhibitory effect on the diffusion and exchange behavior of surfactant molecules at the interface. The surfactant molecules that originally lost the “lateral bondage” are re-affected by the “longitudinal draw” on the oil side, and the diffusion exchange becomes more difficult to occur. The value of *ω*_1_ is significantly lower than that of the gas–liquid interface.

In addition, by comparing the *ω*_1_ values in Figure 2A,B, we found that for the oil-water interfacial film, the *ω*_1_ of C_8_C_10_ with a longer alkyl chain in the hydroxyl para position is always slightly smaller than C_8_C_8_ with a relatively short chain length in the whole concentration range, which fully demonstrates the strong interaction between the long alkyl chain in the hydroxyl para position and the oil molecules.

### 2.3. Effect of Surfactant Concentration on ω_0_

**Figure 3:**
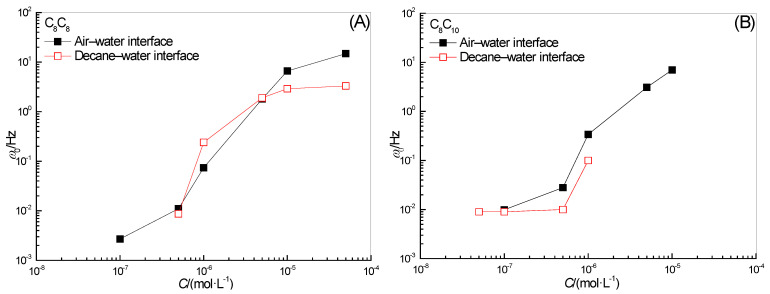
Concentration dependence of $\omega_{0}$ for surfactant solutions at water-air (**A**) and water-decane interfaces (**B**).

The effect of surfactant concentration on *ω*_0_ in the full-frequency spectrum of surface-interface dilational elasticity is shown in Figure 3. The dilational elastic value reaches the platform, which means that the molecules forming the interface film do not exchange with the bulk phase during the perturbation process; at the same time, the molecules on the interface cannot dissipate energy through orientation changes. It should be pointed out that the relaxation processes near the interface film all have their characteristic frequencies, and the frequency *ω*_0_ corresponding to the limit dilational elasticity must be greater than all the characteristic frequencies. The dynamic curve in Figure 3 shows that for C_8_C_8_ and C_8_C_10_, *ω*_0_ increases with the increase in concentration, whether it is adsorbed at the gas–liquid interface or the oil–water interface. The increase in concentration first leads to the intensification of diffusion exchange behavior between bulk phase and interface. At the same time, for the surfactant molecules on the interface, the increase in adsorption quantity means that the molecular orientation is more and more prone to change, and the change of conformation provides a new relaxation process for the system. Therefore, for a group of homologues with similar interface behavior, with the increase in surfactant concentration, the frequency corresponding to the limit dilational elasticity must be higher and higher. In addition, for C_8_C_8_, the value of *ω*_0_ at the oil–water interface at high concentration begins to become lower than that at the gas–liquid interface, which seems to imply the weakening of intermolecular interaction on the surface film; the specific reason will be discussed in detail in the next section.

### 2.4. Effect of Surfactant Concentration on ε_0_

**Figure 4:**
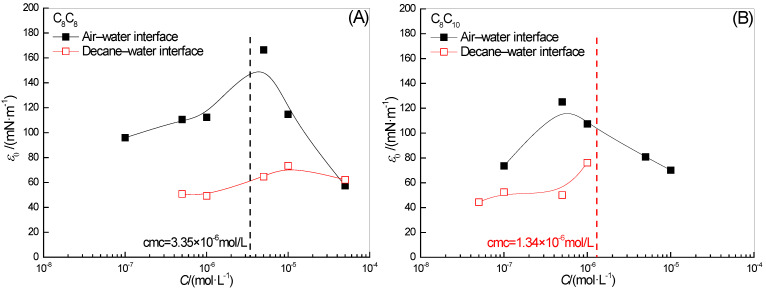
Concentration dependence of limiting dilational elasticity ($\varepsilon_{o}$) of surfactant solutions at the water-air (**A**) and water-decane interfaces (**B**).

The effect of two surfactant concentrations on *ε*_0_ in the full-frequency spectrum of the surface-interface dilational elasticity is shown in Figure 4. With the increase in concentration, the dilational elasticity of two hydroxyl-substituted alkylbenzene sulfonates at the gas–liquid interface will pass a maximum value. In our previous study of dilational rheology by using the periodic oscillation method [39], we found that with the increase in the number of adsorbed molecules on the surface, the orientation of the hydroxyl ortho long-chain alkyl will gradually change from being tiled along the surface to extending into the air. This change in molecular orientation weakens the structure of the surface adsorption film, resulting in a decrease in *ε*_0_. In the previous section on the effect of surfactant concentration on *ω*_1_, we see a slowing of the upward trend in the high concentration range in Figure 3A, which once again confirms the strong interaction between hydrophobic alkyl and the oil phase at the oil–water interface. That is, with the disappearance of the “positioning” effect of hydroxyl and sulfonic acid groups at the gas–liquid interface, the diffusion exchange behavior of surfactants began to intensify, but the presence of oil molecules maintained the viscoelasticity of the interfacial film.

That is to say, our understanding of C_10_C_8_ (Sodium 2-hydroxy-3-decyl-5-octylbenzenesulfonate) through the small low-frequency oscillation is consistent with the conclusion drawn by the interfacial tension relaxation method in this paper: the longer the alkyl chain of the hydroxyl group on the benzene ring, the smaller the elastic modulus of the surfactant molecule at the gas–liquid interface and the greater the attraction of the oil phase side at the oil–water interface.

C_8_C_8_ and C_8_C_10_ are homologues, and the length of the alkyl chain adjacent to their hydroxyl groups is exactly the same, so their limit dilational elasticity values are high. The difference is that the concentration of the transition of the C_8_C_8_ system is more delayed, and the *ε*_0_ value that can be achieved is higher, which may be caused by the difference in the length of the hydroxyl para-alkyl chain. The longer the length of the para-alkyl chain, the more unfavorable to the tile of the para-alkyl on the gas–liquid interface, and the positioning effect is less stable. For the interfacial adsorption film, the stacking structure formed between the alkyl chains is weakened, which is mainly controlled by the behavior of single molecules, the structural similarity leads to the close *ε*_0_ values of C_8_C_8_ and C_8_C_10_. Although the strong interaction between the oil phase and the double-chain alkyl of the surfactant maintains the stability of the surfactant molecules at the interface, the avoidance of diffusion exchange does not mean the continuation of the intermolecular interaction. Compared with the limit dilational elasticity of the surfactant molecules at the gas–liquid interface and oil–water interface, the value at the oil–water interface is about 1/3 of that at the gas–liquid interface. The intermolecular interaction formed by the adjacent alkyl chains of hydroxyl group is indeed destroyed by the insertion of oil molecules.

### 2.5. Full-Frequency Spectrum of Interfacial Dilational Viscosity of Adsorption Film

Viscosity is another important parameter to characterize the characteristics of interfacial adsorption film, which is directly related to the characteristic frequency of the relaxation process. Viscosity modulus is the sum of all relaxation processes occurring at and near the interface, including the diffusion exchange of surfactant molecules between the interface and the bulk phase, the single molecule reorientation of the surfactant at the interface and the rearrangement of a large number of molecules. Among them, the existence of diffusion exchange will weaken the film strength, while other relaxation processes in the film can promote the film strength. Moreover, in addition to the insoluble film formed by the protein, the adsorption film formed by all surfactants has at least a diffusion exchange effect. Therefore, the viscosity must exist for the conventional surface-interface or oil–water system.

**Figure 5:**
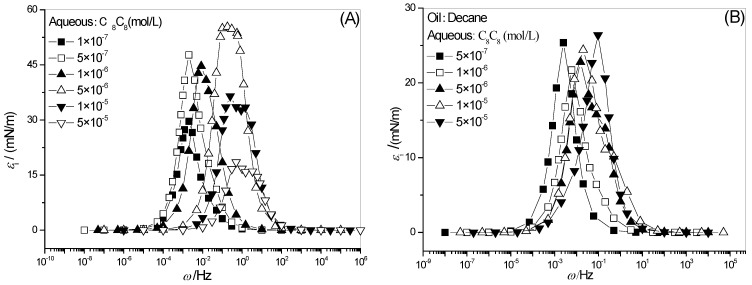
Frequency spectra of surface (**A**) and interfacial (**B**) dilational viscosity for C_8_C_8_ solutions.

Figure 5 shows the full-frequency spectrum of the dilational viscosity of hydroxyl-substituted alkylbenzene sulfonate C_8_C_8_ with different concentrations, where (A) is the gas–liquid interface and (B) is the oil–water interface. It can be clearly seen that whether it is the gas–liquid interface or the oil–water interface, the viscosity curve with frequency will have a maximum value *ε_i_*_0_. The frequency corresponding to the maximum value is the characteristic frequency *ω_i_* of the relaxation process [45]. For a single viscous full-frequency spectrum curve, the number of relative maximum values characterizes the type of relaxation process; there are several maximum values, which indicate how many characteristic frequencies correspond. All the dynamic curves in Figure 5 have only one maximum value, indicating that the properties of the interfacial film are mainly controlled by a relaxation process, whether it is the gas–liquid interface or the oil–water interface.

### 2.6. Effect of Surfactant Concentration on ω_i_

**Figure 6:**
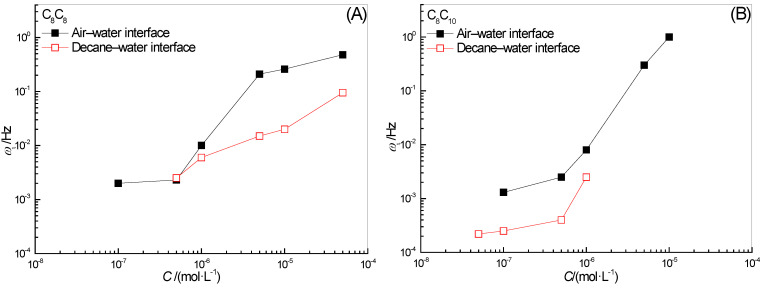
Concentration dependence of the corresponding frequency to the maximum of dilational viscosity for surfactant solutions at water–air (**A**) and water–decane interfaces (**B**).

The effect of surfactant concentration on the frequency *ω_i_* corresponding to the maximum surface or interface dilational viscosity is shown in Figure 6. Analyzing the dynamic curve of the effect of concentration on the characteristic frequency can clarify the relaxation process of the main control interface film properties. Figure 6 shows that, similar to *ω*_1_ and *ω*_0_ which characterize the interfacial dilational elasticity, the characteristic frequency *ω_i_* also shows a rising trend with the increase in surfactant concentration. For the dilational viscosity, the reason for this situation can be attributed to the change of the relaxation process that controls the properties of the film, from the slow relaxation process such as interface rearrangement to the fast diffusion exchange process. At the same time, with the increase in surfactant concentration, the characteristic frequency of diffusion exchange is also accelerated.

For C_8_C_8_ and C_8_C_10_, the *ω_i_* at the oil–water interface is significantly lower than that at the gas–liquid interface in the whole experimental concentration range, which corresponds to the difference in limit dilational elasticity of surfactants at the gas–liquid interface and the oil–water interface. The interaction between the hydroxyl ortho alkyl chains increases the elastic modulus and also introduces the slow relaxation process. The entanglement between molecules will more easily cause molecular orientation and rearrangement. Due to the same length of the hydroxyl ortho alkyl chain, the characteristic frequencies of the two surfactants at the gas–liquid interface are not much different. With the insertion of oil molecules, the effect of the oil phase side on the hydrophobic group not only slows down the diffusion exchange but also slows down the interfacial rearrangement of surfactant molecules, and the value of *ω_i_* decreases sharply. At the same time, C_8_C_10_ with a longer hydrophobic alkyl chain is more attractive to the oil phase than C_8_C_8_, and the characteristic frequency at the same concentration should be smaller in theory; experimental results presented in Figure 6 confirm our idea.

### 2.7. Effect of Surfactant Concentration on ε_i0_

**Figure 7:**
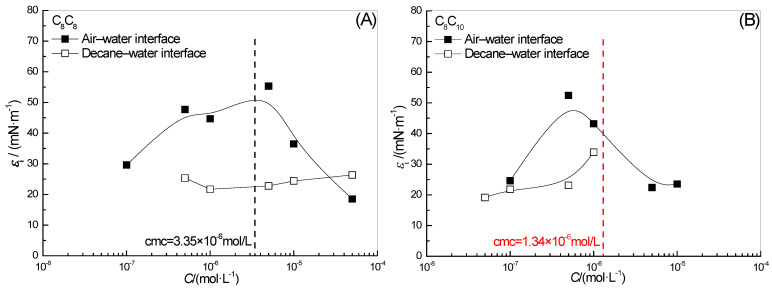
Concentration dependence of the maximum of dilational viscosity of surfactant solutions at the water-air (**A**) and water-decane interfaces (**B**).

The effect of surfactant concentration on the maximum surface-interface dilational viscosity *ε_i_*_0_ is shown in Figure 7. In comparing the effect of surfactant concentration on the maximum dilational elasticity *ε*_0_ in Figure 4, we find that the variation trend of the maximum value for dilational viscosity is similar to the trend of dilational elasticity, and the corresponding concentration is also almost the same when the modulus begins to appear. As mentioned above, the positioning effect of the double hydrophilic groups leads to the entanglement of hydroxyl ortho alkyl chains between different surfactant molecules at the interface; the moment when the intermolecular interaction is the strongest is also the moment when the relaxation processes such as interface rearrangement are introduced most: they are interdependent and complementary. As for the oil–water interface, the insertion of oil molecules weakens the interaction between molecules, the total energy in the interface film decreases and the energy available for dissipation in the system decreases accordingly. Therefore, for C_8_C_8_ and C_8_C_10_, the viscous modulus at the oil–water interface is also much smaller than that at the gas–liquid interface in all concentration ranges.

**Figure 8:**
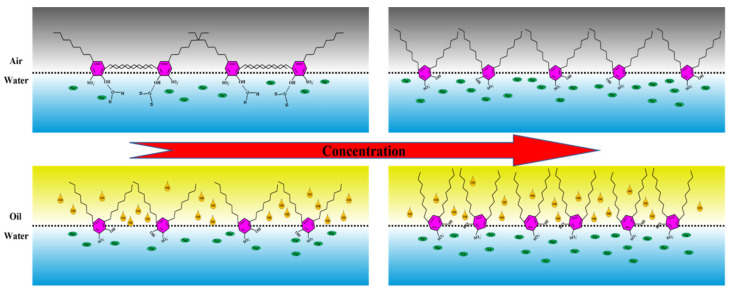
The molecular arrangement diagram of hydroxyl-substituted alkylbenzene sulfonate C_8_C_8_ at the gas–liquid interface and oil–water interface.

The variation curve of the maximum viscosity modulus with the concentration makes us clearly realize that for hydroxyl-substituted alkylbenzene sulfonates, the dominant factor of film properties is the length of the hydroxyl ortho alkyl chain, which has little relationship with the length of the alkyl chain on the hydroxyl para position. C_8_C_8_ and C_8_C_10_ have the same length of hydroxyl ortho alkyl chain, which corresponds to the same maximum viscosity modulus at the gas–liquid interface. In addition, we have also investigated the surface dilational rheology properties of the same series of C_10_C_10_ (Sodium 2-hydroxy-3,5-didecylbenzenesulfonate). The maximum dilational modulus can reach 300 mN/m [46], which is significantly larger than the two compounds with shorter hydroxyl ortho alkyl chains in this paper. Combined with the previous experimental results, we take C_8_C_8_ as an example to give a molecular arrangement diagram for hydroxy-substituted alkylbenzene sulfonates at the surface-interface, as shown in Figure 8.

At the gas–liquid interface, the alkyl chain adjacent to the hydroxyl group of the C_8_C_8_ molecule will produce strong intermolecular interactions under the dual positioning of the sulfonic acid group and the hydroxyl group. At this time, the properties of the interface film are mainly controlled by the slow relaxation process such as interface rearrangement. With the further increase in the surfactant concentration, the ortho alkyl group begins to gradually extend to the air and the relaxation process begins to be dominated by diffusion exchange. On the other hand, at the oil–water interface, oil molecules will destroy the interaction between the adjacent alkyl groups and hydroxyl groups. The properties of the interface film have always been controlled by single-molecule fast relaxation processes such as diffusion exchange. However, with the increase in adsorption quantity on the interface, the alkyl chain is more and more affected by the oil phase, which will increase the difficulty of diffusion exchange to a certain extent and the characteristic frequency will be slightly reduced at high concentration.

It should be pointed out that the above molecular arrangement diagram is obtained when distilled water is used as the solvent, while in the actual application process, a certain ionic strength is often retained in the aqueous phase. For hydroxyl-substituted alkylbenzene sulfonates, the presence of ionic strength often produces electrostatic shielding and enhances the intermolecular interaction on the surface-interface. Therefore, whether it is the gas–liquid interface or the oil–water interface, the characteristic time of the slow relaxation process is likely to become longer and change the main controlling factor of the interfacial film properties.

## 3. Experiment Section

### 3.1. Materials

**Figure 9:**
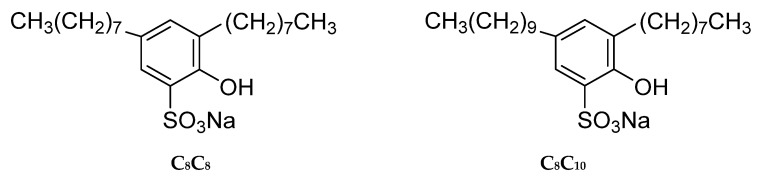
The structures and abbreviations of hydroxy-substituted alkyl benzene sulfonates.

Hydroxyl substituted sodium alkylbenzene sulfonate, 2-hydroxy-3-octyl-5-octyl benzene sulfonate (C_8_C_8_OHphSO_3_Na) and 2-hydroxy-3-octyl-5-decyl benzene sulfonate (C_8_C_10_OHphSO_3_Na) were all self-made in our laboratory and the purity was more than 99%. The critical micelle concentrations (cmc) were 3.35 × 10^−6^ mol/L and 1.34 × 10^−6^ mol/L, respectively. The surface tension at cmc was about 26.89 mN/m and 27.01 mN/m, respectively. The molecular structure is shown in Figure 9, the molecular weights were 420 and 448, respectively. The active fractions in crude oil will also diffuse and exchange between the oil phase and the oil–water interface during the test; in order to avoid the interference of their presence on the oil–water interface behavior of surfactant molecules, *n*-alkane was selected as the oil phase in this paper. *N*-decane was purchased from Tianjin Bondi Chemical Co., Ltd. (Tianjin, China). The experimental water is deionized water after redistilled, and the resistivity is ≥18 MΩ·cm.

### 3.2. Experimental Method

During the experiment, the TRACKER interface dilational rheometer [47] produced by IT-CONCEPT company in France was used for the viscoelastic test. After the system is completely balanced, the surface/interfacial tension relaxation experiment is carried out by measuring the surface/interfacial tension response after a sudden disturbance for the suspended bubble/droplet by using the drop shape analysis method [48]. The disturbance amplitude here is 15% and the time of instantaneous dilational is ≤0.7 s. Because the characteristic frequency of the fast relaxation process, such as single molecule orientation, change on the interface is often in the order of 10^−1^ Hz; in order for all the interface behavior to be observable in the experiments, the shorter the time of the instantaneous disturbance is, the better. The experimental temperature was controlled at 30.0 ± 0.5 °C. The aqueous phase was C_8_C_8_ and C_8_C_10_ solutions with different concentrations prepared by secondary distilled water and the oil phase was n-decane.

### 3.3. Theoretical Background

For a real system with multiple relaxation processes, due to the additivity of the relaxation process, the decay curve can be expressed by the sum of multiple exponential equations, such as Formula (2) [49]: where *τ_i_* is the characteristic frequency of the ith process, Δγ*_i_* is the contribution of the ith process and *n* is the number of total processes.

(2) $$\Delta \gamma =\overset{n}{\underset{i=1}{\sum }}\Delta \gamma_{i}exp\left(-\tau_{i}t\right)$$

For an instantaneous interface deformation process with amplitude Δ*A*, after the deformation stops, the value *ε*(*ω*) of the dilational frequency modulus at a certain frequency can be obtained by Fourier transform of the interfacial tension decay curve, as shown in Formula (3) [11,12]:

(3) $$\varepsilon \left(\omega \right)=\frac{FT\Delta \gamma \left(t\right)}{FT\left(\Delta A/A\right)\left(t\right)}=\frac{\int_{0}^{\infty }\Delta \gamma \left(t\right)exp\left(-i\omega t\right)dt}{\int_{0}^{\infty }\left[\Delta A\left(t\right)/A\right]exp\left(-i\omega t\right)dt}$$

By introducing Formula (2) into Formula (3), the calculation formulas of interface dilational elasticity (*ε_d_*) and dilational viscosity (*ε_i_*) under different frequency conditions can be obtained by deformation, such as Formulas (4) and (5) [11,12]:

(4) $$\varepsilon_{d}\left(\omega \right)=\frac{\omega }{\Delta A/A}\overset{\infty }{\underset{0}{\int }}\Delta \gamma \left(t\right)\sin\left(\omega t\right)dt$$

(5) $$\omega \eta_{d}\left(\omega \right)=\frac{\omega }{\Delta A/A}\overset{\infty }{\underset{0}{\int }}\Delta \gamma \left(t\right)\cos\left(\omega t\right)dt$$

## 4. Conclusions

In this paper, the interfacial tension relaxation method was used to explore the adsorption behavior of two hydroxyl-substituted alkylbenzene sulfonates C_8_C_8_ and C_8_C_10_ at the gas–liquid interface and oil–water interface with the same length of the hydroxyl ortho alkyl chain and different length of the para-alkyl chain. The main controlling factors of surface-interface film properties under different conditions were clarified and the following conclusions were obtained:

(1) The sulfonic acid group and hydroxyl group on the benzene ring all interact with the water phase, which will have a certain “positioning” effect on the interfacial configuration of the surfactant molecules. The long-chain alkyl groups adjacent to the hydroxyl group tend to extend along the interface, showing strong intermolecular interaction; while the long-chain alkyl at the para position of the hydroxyl group tends to extend into the air. Therefore, the length of the hydroxyl ortho alkyl chain is the main controlling factor of the film properties and has little to do with the length of the para-alkyl chain.
(2) For the gas–liquid interface, at first, due to the interaction between alkyl chains, the properties of the interfacial film are mainly controlled by the slow relaxation process of interfacial rearrangement. With the further increase in surfactant concentration, the adjacent alkyl groups and hydroxyl groups begin to extend to the air, and the interfacial film begins to be controlled by the fast relaxation process that is dominated by diffusion exchange.
(3) For the oil–water interface, the insertion of oil molecules will destroy the interaction between alkyl chains. The properties of the interfacial film have always been controlled by the fast relaxation process that is dominated by diffusion exchange. With the further increase in surfactant concentration, the interaction between alkyl chains and oil phase is enhanced, and the characteristic frequency of the fast relaxation process will be slightly reduced.

## Data Availability

Due to privacy temporarily do not share data.

## Sample Availability

Samples of the compounds C_8_C_8_ and C_8_C_10_ are available from the authors.
